# Automatic labels are as effective as manual labels in digital pathology images classification with deep learning

**DOI:** 10.1016/j.jpi.2025.100462

**Published:** 2025-07-22

**Authors:** Niccolo Marini, Stefano Marchesin, Lluis Borras Ferris, Simon Püttmann, Marek Wodzinski, Riccardo Fratti, Damian Podareanu, Alessandro Caputo, Svetla Boytcheva, Simona Vatrano, Filippo Fraggetta, Iris Nagtegaal, Gianmaria Silvello, Manfredo Atzori, Henning Müller

**Affiliations:** a Information Systems Institute, University of Applied Sciences Western Switzerland (HES-SO Valais), Sierre, Switzerland; b Department of Information Engineering, University of Padua, Padua, Italy; c University of Applied Sciences and Arts Dortmund, Dortmund, Germany; d Department of Measurement and Electronics, AGH University of Krakow, Krakow, Poland; e SURFsara, Amsterdam, the Netherlands; f Department of Pathology, Ruggi University Hospital, Salerno, Italy; g Pathology Unit, Gravina Hospital Caltagirone ASP, Catania, Italy; h Ontotext, Sofia, Bulgaria; i Institute of Information and Communication Technologies, Bulgarian Academy of Sciences, Sofia, Bulgaria; j Department of Pathology, Radboud University Medical Center, Nijmegen, the Netherlands; k Department of Neurosciences, University of Padua, Padua, Italy; l Medical faculty, University of Geneva, 1211 Geneva, Switzerland

**Keywords:** Automatic weak labels, Deep learning, Histopathology image classification, Noisy labels

## Abstract

The increasing availability of biomedical data is helping to design more robust deep learning (DL) algorithms to analyze biomedical samples. Currently, one of the main limitations to training DL algorithms to perform a specific task is the need for medical experts to manually label the data. Automatic methods to label data exist; however, automatic labels can be noisy, and it is not completely clear in which situations they can be used to train DL models.

This paper aims to investigate under which circumstances automatic labels can be used to train a DL model for the classification of whole slide images. The analysis involves multiple architectures, such as convolutional neural networks and vision transformer, and 10,604 WSIs as training data, collected from three use cases: celiac disease, lung cancer, and colon cancer, which include respectively binary, multiclass, and multilabel data. The results identify 10% as the percentage of noisy labels before a performance drop-off, so to train effective models for the classification of WSIs, reaching, respectively, F1-scores of 0.906, 0.757, and 0.833. Therefore, an algorithm generating automatic labels needs to stay within this range to be adopted, as shown by the application of Semantic Knowledge Extractor Tool as a tool to automatically extract concepts and use them as labels. Automatic labels are as effective as manual labels in this case, achieving solid performance comparable to that obtained by training models with manual labels.

## Introduction

### Background

Developing deep learning (DL) algorithms fosters the design of new tools that can be trained on clinical data without human intervention, especially in domains where annotations are expensive, such as histopathology. Histopathology is the gold-standard to diagnose cancer.^10^^,^^26^ The domain involves the analysis of small tissue slices to identify microscopic findings related to dangerous diseases,^16^ such as cancer. Tissue slices undergo microscopic examination by a medical expert named a pathologist, who usually needs several minutes to analyze a single sample.^25^ Despite the increasing digitization of tissue samples, histopathological samples are not always analyzed exploiting digital aid in clinical practice.^13^^,^^14^ Digital pathology is a domain involving the management and digitization of tissue specimens, called whole slide images (WSI). WSIs are high-resolution images, usually stored in a pyramidal format, to capture different magnification levels of details.^38^ Usually, the highest resolution levels result in a spatial high-resolution of 0.25–0.5 μm per pixel, corresponding to an optical magnification of 20×–40×. WSIs are usually associated with written pathology reports. Pathology reports are most often semi-structured free-text documents containing information about the patient's anamnesis, the tissue specimen type, and the findings and observations identified by a pathologist during the tissue examination.^18^^,^^20^ WSIs and reports are usually stored in the laboratory information system (LIS), which easily enables sample retrieval. The increasing collection of biomedical samples encourages the design of automatic tools to analyze WSIs in the computational pathology domain.^26^^,^^28^^,^^30^ Most of the algorithms are currently based on DL, such as CNNs (convolutional neural networks) or ViT (visual transformers).^9^^,^^51^

Even if computational pathology algorithms show accurate and robust performance in tasks such as WSI classification or segmentation, several challenges are still open, such as data labels.^1^^,^^5^^,^^7^^,^^26^^,^^30^^,^^34^ Labels are required to train supervised learning algorithms. However, the collection of labels is not trivial, considering both strong and weak annotations. Even if strong labels (i.e., pixel-wise annotations) usually achieve the most accurate performance when training a DL model, they require a pathologist to analyze samples, which can be time-consuming and is often unfeasible.^24^ Therefore, the research on the analysis of WSIs is most frequently based on the exploitation of weak (i.e., image-level) labels. Weak labels are related to the global image, even if they are most frequently based on a small region of the image, including specific characteristics, such as cancer.^11^ Weak labels are inherently more noisy than pixel-wise annotations because the regions leading to a specific label may be a small percentage of the entire image (e.g., 1–2%). For this reason, algorithms based on weak labels require larger training datasets to reach high performance. Currently, most weakly supervised algorithms in computational pathology are based on the multiple instance learning (MIL) approach^6^ that models the whole image as a bag of instances, where only global annotations are available. An MIL approach includes several algorithms that recently showed high performance when adopted on large-scale datasets.^5^^,^^7^^,^^19^^,^^21^^,^^29^^,^^49^ For example, Campanella et al.^5^ showed that it is possible to reach almost perfect predictions on binary classification (cancer vs. non-cancer) using around 10,000 weakly annotated WSIs on 3 use cases: skin, breast, and prostate cancer. Weak labels are produced much faster than strong labels, because they can be extracted from reports more or less automatically. For example, analyzing a report may take approximately 30 s–1 min, compared with analyzing an entire image, which easily takes around 30 min. However, human intervention is usually still required to analyze reports unless the LIS, where the samples and corresponding reports are stored, has a specific structure to retrieve structured data automatically according to the characteristics that can be used as labels. Unfortunately, most LISs currently do not have this feature because they are organized around the general workflow and documents rather than structure data.

Automatic methods for extracting concepts from reports and using them as weak labels already exist,^33^ but the noise of weak labels can make automatic labeling challenging. This paper investigates under which circumstances automatic labels (i.e., labels automatically generated by an algorithm) can be adopted to train DL models, alleviating the need for experts to manually annotate data. In particular, the goal is to identify when the performance achieved using this type of label reaches results comparable to those obtained using manual labels (i.e., labels produced by a medical expert) so that data included in LISs can be fully exploited to build more robust and accurate tools to diagnose diseases. The characteristics investigated in the paper involve the percentage of incorrectly annotated, automatic labels that is possible while still reaching comparable performance as manual labels, the nature of labels (e.g., binary, multiclass, and multilabel), and the DL architecture (robust or less robust to noise). Incorrectly extracted automatic labels are annotations that are automatically produced by an algorithm and do not match the ground truth, where the ground truth is manually created.

### Contribution

The paper includes a comparison of DL architectures trained with automatic and manual labels for the classification of WSIs. Manual labels are randomly modified with varying percentages of noise, simulating the output of an algorithm generating automatic labels with different numbers of errors (i.e., a varying percentage of noise). The purpose of the comparison is to assess the robustness of DL architectures to noisy labels that can result from data annotation performed by an automated approach. The random perturbation involves modifying the labels. In the celiac disease (CD) use case, labels are flipped because the dataset includes binary annotations. A different class is assigned to a sample in the lung cancer use case because the dataset includes multiclass annotations. In the colon cancer use case, the modifications involve one or more classes for every sample because the dataset includes multilabel annotations. Together with the random perturbation of training labels, the architectures are trained also with labels provided by the Semantic Knowledge Extractor Tool (SKET).^31^ SKET is a tool used to automatically extract meaningful semantic concepts from reports that are weak labels for the corresponding samples. SKET is adopted to evaluate the behaviour of models when trained with automatically annotated data, aiming to confirm the findings identified with noisy labels.

The analysis involves three use cases with differnt tissue types: CD, lung cancer, and colon cancer, composing a training dataset with over 10,000 WSIs, used to train 3 DL architectures: CLAM,^29^ transMIL,^45^ and ViT.^7^

**Fig. 1:**
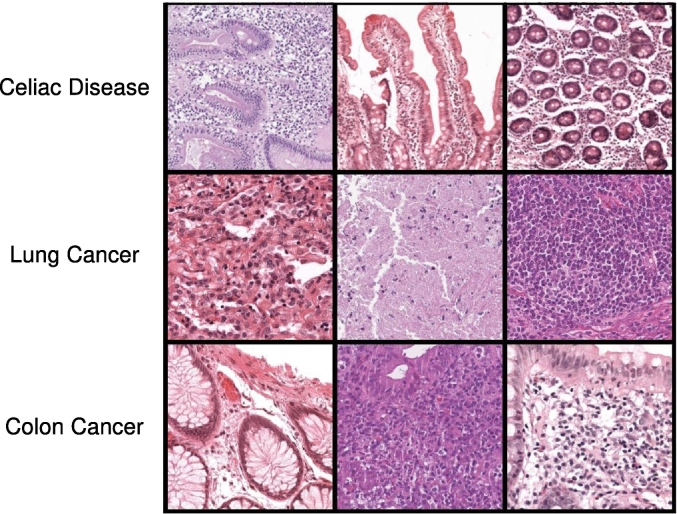
Overview of the use cases analyzed in the paper. The upper line includes examples of duodenal tissue samples related to celiac disease. The central line includes examples of lung tissue samples. The bottom line includes examples of colon tissue.

CD is an autoimmune disorder leading to damage in the small intestine, resulting in a range of gastrointestinal and systemic symptoms.^4^ Globally, CD affects about 1–2% of the population,^27^ with variations across regions. In particular, the examination of biopsies aims to identify villous atrophy, crypt hyperplasia, and increased intraepithelial lymphocytes. This paper labels duodenal samples with CD or as normal tissue (binary labels). Lung cancer is the leading cause of death related to cancer worldwide.^42^^,^^44^ It is often categorized into two main primary groups: non-small cell lung cancer (NSCLC), which represents the large majority of cases (about 85% of cases), and small-cell lung cancer (SCLC), which is less common, but more aggressive. Furthermore, NSCLC is further described with subtypes, such as LUng ADenocarcinoma (LUAD), LUng Squamous cell Carcinoma (LUSC). Diagnosis of lung cancer through biopsies often involves the identification of irregular cell patterns, architectural distortion, and increased cellular density.^47^ In this paper, lung samples are labeled with SCLC, LUAD, LUSC, and normal tissue. Colon cancer is the fourth most frequently diagnosed cancer worldwide.^2^ Colon cancer diagnosis involves the identification of multiple concepts, such as the presence of cancer and the evaluation of polyp shapes and possible abnormalities leading to dysplasia. In this paper, colon samples are labeled with colon cancer, high-grade dysplasia (HGD), low-grade dysplasia (LGD), hyperplastic polyp, and normal tissue (multilabel labels). Fig. 1 shows some histopathology samples corresponding to the three tissue types.

## Materials and methods

### Dataset composition

The dataset used in this paper includes WSIs and reports (paired together) of CD, lung cancer, and colon cancer collected from two hospitals: the Catania cohort and from Radboud University Medical Center (RUMC). WSIs are used to train and test several computer vision architectures on image-level classification. WSIs are gigapixel images, including tissue samples, that can exhibit important heterogeneity, for example in terms of staining.^32^^,^^36^ Image heterogeneity is a consequence of different acquisition procedures across labs related to the chemical reagents applied to the specimen, the environment (temperature) and to the slide scanners as a whole. One of the main consequences of the heterogeneity is the stain variability, leading to different color variations, intensity and uniformity of stains across different slides (as shown in Fig. 1). The WSIs collected in this dataset also show heterogeneity, aiming to replicate a common scenario in digital pathology. WSIs collected from the Catania cohort were scanned with two 3DHistech scanners and two Aperio scanners and stored with a magnification of 20×–40×; WSIs collected from RUMC were scanned using 3DHistech scanners, mainly stored at 40× magnification.

Reports are used to extract meaningful concepts used as weak automatic labels to train the model to classify WSIs. Reports include free-text descriptions summarizing the findings from tissue examination. The findings are reported in a field named *Conclusion*, containing either macroscopic or microscopic observations. Even if a report includes many fields, only the findings are relevant for the analysis proposed in the paper. Therefore, additional patient information, such as family history or personal data, is discarded in our case. Text reports show heterogeneity, mainly related to the source language and the text content. Reports are collected from an Italian and a Dutch hospital, therefore they have to be translated into English, to standardize the analysis. The text content slightly differs across sources because the Catania reports are related to a single slide, whereas the RUMC reports include a specific field for the findings identified in a tissue block, which may encompass multiple slides related to several images. The text content slightly differs across sources because the Catania reports contain a field specifically for the findings identified in a single slide, whereas the RUMC reports include a field specifically for the findings identified in a tissue block, which may encompass multiple slides. Therefore, RUMC reports needed a pre-processing step to separate the content and link it to the corresponding WSI. Furthermore, samples are collected over several years and reports are produced by many different pathologists, each adopting a unique style of writing.

The dataset includes samples collected from three different use cases: CD^43^, lung cancer,^12^ and colon cancer.^33^ Data are randomly selected from the LISs to be in a real scenario. The goal is to show that the approach can be generalized to different types of tissue (both in terms of images and reports). Different labels are used: CD samples are annotated with binary labels, lung samples with multiclass labels, and colon samples with multilabel samples. Table 1 includes a detailed composition of data related to CD collected from pathology reports, split into training and testing partitions. Data are labeled with binary labels: CD and normal tissue. Table 2 includes a detailed composition of data related to lung cancer collected from pathology reports, split into training and testing partitions. Data are labeled with multiclass labels: small-cell cancer, non-small adenocarcinoma cell cancer, non-small squamous cell cancer, and normal tissue. Table 3 includes a detailed composition of data related to colon cancer collected from pathology reports, split into training and testing partitions. Data are labeled with multilabel labels: adenocarcinoma, HGD, LGD, hyperplastic polyp, and normal tissue.

**Table 1:** Composition of the samples related to the celiac disease use case, considering automatically generated labels (automatic labels) and ground-truth labels (manual labels). Data are labeled with binary labels: celiac disease and normal tissue. The dataset is split into training and testing partitions. The model is trained and validated, adopting a 10-fold cross-validation approach.

Source	Celiac disease	Normal tissue	Total
*Training dataset: Automatic labels*			
Catania	47	711	758
RUMC	217	524	741
Total	264	1235	1499

*Training dataset: Manual labels*			
Catania	61	697	758
RUMC	223	518	741
Total	284	1235	1499

*Testing dataset*			
Catania	10	83	93
RUMC	37	63	100
Total	47	146	193

**Table 2:** Composition of the samples related to the lung cancer use case, considering automatically generated labels (automatic labels) and ground-truth labels (manual labels). Data are labeled with multiclass labels: Small-cell cancer, non-small adenocarcinoma cell cancer, non-small squamous cell cancer, and normal tissue. The dataset is split into training and testing partitions. The model is trained and validated, adopting a 10-fold cross-validation approach.

Source	SCLC	LUAD	LUSC	Normal	Total
*Training dataset: Automatic labels*					
Catania	49	526	250	226	1051
RUMC	1	262	195	1041	1499
Total	50	788	445	1267	2550

*Training dataset: Manual labels*					
Catania	50	519	271	211	1051
RUMC	1	260	173	1065	1499
Total	51	779	444	1276	2550

*Testing dataset*					
Catania	12	62	67	32	173
RUMC	0	55	29	110	194
Total	12	117	96	142	367

**Table 3:** Composition of the samples related to the colon cancer use case, considering automatically generated labels (automatic labels) and ground-truth labels (manual labels). Data are labeled with multilabel annotations: adenocarcinoma, high-grade dysplasia (HGD), low-grade dysplasia (LGD), hyperplastic polyp, and normal tissue. Due to the multilabel nature of labels, the total samples for each class may not correspond to the total number of samples. The dataset is split into training and testing partitions. The model is trained and validated adopting a 10-fold cross-validation approach.

Source	Adenocarcinoma	HGD	LGD	Hyperplastic	Normal	Total
*Training dataset: Automatic labels*						
Catania	776	761	1288	511	596	3095
RUMC	383	377	853	943	1341	3460
Total	1159	1138	2141	1454	1937	6555

*Training dataset: Manual labels*						
Catania	865	774	1273	535	570	3095
RUMC	394	362	878	965	1309	3460
Total	1259	1136	2151	1500	1879	6555

*Testing dataset*						
Catania	111	96	113	32	98	348
RUMC	75	65	146	119	193	520
Total	186	161	259	151	291	868

### Data analysis pipeline

The training schema is based on computer vision algorithms to classify WSIs, comparing the performance of automatic and manual labels during the training. Those algorithms are based on weak labels because they are easier to collect, even if they still require the intervention of medical experts. This paper adopts three different MIL backbones: two CNNs, CLAM and transMIL, and a ViT. The architectures are trained to evaluate the effect that automatic labels may have on the training of models to classify WSIs. First, they are trained with noisy labels, randomly generated to perturb the manual labels with a different percentage (1%, 2%, 5%, 10%, 20%, and 50%) of noise. This experiment's goal is to evaluate the effect that different amounts noisy labels have on the performance of a model. The application of random perturbations to the label allows to alleviate any hypothesis on the nature of labeling errors. Due to the nature of reports, not all samples are equally likely to be mislabeled. Consider weak labels inferred by medical reports: some reports, due to their content, may be more easily mislabeled, for example, when the textual description of findings is wordy and includes high-level details or ambiguous findings. In principle, it is not simple to design the possible difficulties occurring in analyzing the report, because they strongly depend on the structure of the report, on the organization of the findings, on the level of details expressed by the medical expert and on the clinical exam. On the other hand, a parameter that always influences the performance of DL models besides the report content is the number of mislabeled samples. Therefore, what is relevant to analyze and what this paper targets is the effect that different percentages of mislabeled training data have on algorithms.

**Fig. 2:**
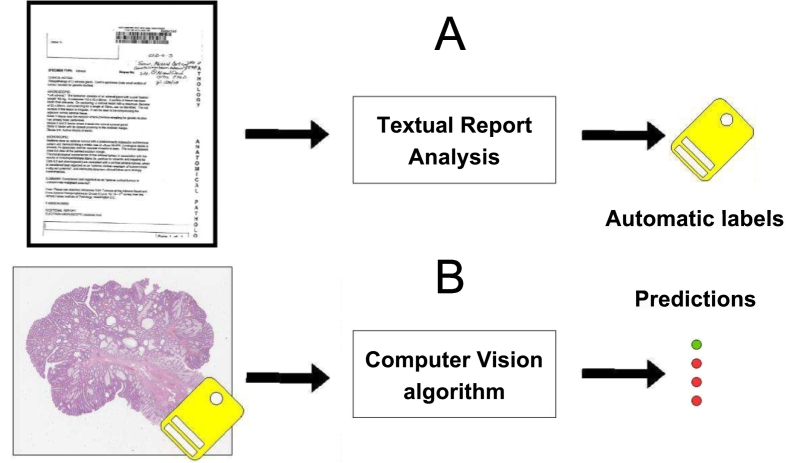
Overview of the data analysis pipeline proposed in the paper. It includes two steps. The first step (A) involves the analysis of text reports to extract meaningful concepts that can be used as weak (automatic) labels for WSIs. The second step (B) involves image analysis through computer vision algorithms that are transparent to the user and can be exchanged to predict the content of the images.

Randomly perturbed annotations are a tool to modeling errors. In order to validate the findings, a real tool to extract concepts from reports is adopted: the SKET.^31^ The goal of its application is to assess a real-world scenario in which a tool to generate automatic labels is adopted, to confirm the validity of the rules on the percentage of mislabeled samples identified with randomly perturbed samples. Fig. 2 shows an overview of the data analysis pipeline.

### Computer vision architectures

The paper compares three computer vision algorithms to classify WSIs as backbones to evaluate the effect of noisy labels on different architectures, including two CNNs and a ViT. The CNNs have a ResNet34 backbone, whereas the ViT has a backbone similar to the one used in Chen et al.,^7^ considering a single magnification level. In both cases, the backbones are designed to output an embedding of size 128 representing a single WSI, so that the same classifier can be adopted for all architectures, modifying the output classes based on the use case.

*CLAM.* Clustering-constrained attention MIL (CLAM)^29^ is a MIL framework based on an attention-based network that highlights relevant regions inside the WSI to improve the WSI-level prediction. CLAM exploits a mechanism on the single instances to aggregate them on clusters, according to the instance similarity, to enrich the WSI representation and reach higher WSI-level predictions. CLAM can have one or more attention branches, depending on the number of classes. In this paper, a single attention branch (CLAM SB) is used when the model is used on CD (binary labels), whereas a multiple attention branch (CLAM MB) is used on the other two use cases.

*transMIL.* transMIL^45^ is a MIL framework developed to exploit the morphological and spatial characteristics of WSIs. Even if morphological and spatial characteristics of images are important, the attention mechanism does not consider them when evaluating input instances. transMIL exploits Transformer architectures^48^ to highlight relationships between single instances, modeling input instances as a sequence of tokens and evaluating the similarity among instances.

*Vision transformer.* A ViT^17^^,^^46^ is a DL architecture to analyze images, adopting the selfattention mechanism to process input data instead of convolutional layers, showing often a better performance in terms of accuracy and efficiency. The architecture processes input data as a sequence of input tokens that are small sub-regions of the input image (usually 16 × 16 pixels). The architecture includes 12 encoder layers producing the embedding to feed the classifier.

### Semantic Knowledge Extractor Tool (SKET)

SKET^31^ is an unsupervised tool combining a rulebased expert system with machine learning models, chosen to extract meaningful concepts from reports and use them as weak labels for WSIs.^31^^,^^37^ The approach includes Named Entity Recognition, Entity Linking and Data Labeling. Named Entity Recognition involves pre-trained models (ScispaCy models^40^), developed to work on biomedical data, and large Word2Vec word vectors^39^ trained on the PubMed Central Open Access Subset.^39^ Entity Linking combines similarity-matching techniques to match ad-hoc concepts to a reference ontology. Data Labeling involves mapping the concepts with a set of annotation classes. SKET is an unsupervised approach; therefore, no training data are required to tune it in our case. This feature is relevant because it does not require data annotation for training, unlike other natural language processing (NLP) algorithms.

## Experimental setup

### Image pre-processing

Image pre-processing includes the WSI splitting into patches. Because of their gigapixel characteristics, WSIs usually do not fit modern GPU hardware memory; therefore, they have to be split into patches. In this paper, WSIs are split 224 × 224 pixel patches using the Multi Scale Tools library.^35^ The choice of the size is related to the characteristics of ResNet34 backbone, requiring fixed input size. Patches are extracted from a 5× magnification, considering celiac samples, whereas lung and colon patches are sampled from a 10× magnification. The magnifications are chosen considering that the magnification allows the identification of peculiar morphological features that are useful for the classification task.The choice of the magnification to examine is driven by the characteristics of the problem to solve: CD diagnosis requires to identify the villous shape and the crypts, therefore 5× magnification is chosen; on the other hand, lung and colon require a more refined level of magnification, because the shape of glands is as relevant as the cell infiltration, therefore 10× is chosen. Not all sampled patches are selected: the ones from background regions are discarded, being not informative. Identifying background regions involves applying HistoQC tool,^22^ which generates tissue masks.

### Report pre-processing

The report pre-processing only involves the translation of all reports into English. Original reports are stored in Italian and Dutch, depending on the workflow from which they are collected. The translation is necessary because state-of-the-art NLP algorithms are mostly developed to work with inputs in English, particularly the tooldsthat we are using. MarianMT neural machine translation models^23^ are used to translate the content of the reports to English. Automatic translation is not perfedt but suffient for the broad concepts we are extracting.

### Architecture pre-training

The backbones of DL algorithms to analyze images are pretrained using self-supervised algorithms: simCLR^8^ for the CNNs (CLAM and transMIL), DINO v2^41^ for the ViT.

Both algorithms are adopted to learn meaningful features from unannotated input data, exploiting similarities and dissimilarities between input samples. In this paper, the input data for the algorithms are the patches sampled from the training partition. Because data are unannotated, no information is available regarding patch similarity. Therefore, data augmentation is adopted: samples are similar to their augmented versions and dissimilar from the other samples within a batch. The algorithms differ in the data augmentation strategy. simCLR is designed for CNNs and its augmentation pipeline includes several operations, applied with a probability of 0.5: random rotations (90/180/270 degrees), vertical/horizontal flipping, hue-saturation-contrast (HUE) color augmentation, RGB shift, color jitter, Gaussian noise, elastic transformation, and grid distortions. DINO is designed for ViT and involves a knowledge distillation mechanism: two networks, a teacher and a student, are involved in the training. The teacher is a larger model producing outputs that the student aims to mimic and replicate. Both models are directly trained with two different augmented versions of input samples. However, the student is also trained with a cropped version (96 × 96 pixels) of the teacher inputs. The DINO v2 augmentation pipeline includes two pipelines: the first one includes color jitter, horizontal/vertical flipping, Gaussian blur, and solarization.

### Image data augmentation pipeline

The augmentation library^3^ is adopted to apply data augmentation to input images. The operations involved are random rotations (90/180/270 degrees), vertical/horizontal flipping and HUE color augmentation. The operations from the data augmentation pipeline are selected with a probability of 0.5 and applied at the image-level, so that all the patches are augmented consistently.

### Metric to evaluate the performance

The performance of the models is evaluated in terms of WSI classification using the weighted F1-score. The classification problem can be defined as binary (CD), multiclass (lung cancer), or multilabel (colon cancer). The F1-score is a metric used to measure the accuracy of a classifier, combining recall and precision. Precision evaluates how robust a classifier is in avoiding predicting negative samples as positive ones, whereas recall evaluates whether all positive samples are well classified. Data may show unbalanced class distribution in all the use cases, because they are randomly selected from workflows, aiming to simulate a realistic scenario. For this reason, a weighted macro F1-score is adopted. The weighted F1-score tackles class imbalance, evaluating the F1-scores for the single classes and then averaging them according to the class support (number of true samples for the class). The weighted F1-score is reported as the average and standard deviation of the 10 experiment repetitions evaluated on the test partition.

### Statistical significance test

The performance difference among different setups is evaluated through the Wilcoxon Rank-Sum test.^50^ The test aims to establish if the results of two different experiments are statistically significantly different (*P*-value <0.05).

### K-fold cross-validation

All the setups presented in the paper are trained using *k*-fold cross-validation to evaluate the model's robustness to the data used for training. The training partition is divided into *k* folders (*k* = 10 in this paper). During every training repetition, k-1 folders are used to train the model, whereas the other group is used to validate it. Data are split into partitions considering the patients so that WSIs collected from a patient cannot be in two different partitions

### Hardware and software

The experiments are developed exploiting Python libraries. The DL algorithms are implemented and trained using PyTorch 2.2.0 and run on a Tesla V100 GPU. WSIs are accessed using openslide 3.4.1.^15^ WSI pre-processing involves the Multi Scale Tools library^35^ and data augmentation is applied using albumentations 1.3.1.^3^ The performance of the model is quantitatively evaluated using the metrics implemented by sci-kit-learn 0.22.

### Hyperparameters

The optimal configuration setup of both CNN and ViT hyperparameters is identified using a grid search algorithm. Considering the validation partition, the optimal set reaches the lowest loss function of the classification of WSIs. The parameters tested with the grid search algorithm are: the batch size (4 selected; 1, 2, 4, and 8 tested); the CNN optimizer (Adam selected); the ViT optimizer (Adam selected; Adam, LARS, and AdamW tested); the number of epochs when the CNN model is trained (15; over this number of epochs, the loss function evaluated on the validation partition no longer decreases); the number of epochs when the HIPT model is trained (15; over this number of epochs, the loss function evaluated on the validation partition no longer decreases); the learning rate (10^*−*4^; 10^*−*2^, 10^*−*3^, 10^*−*4^, and 10^*−*5^ were tested); the decay rate (10^*−*4^; 10^*−*2^, 10^*−*3^, 10^*−*4^, and 10^*−*5^ were tested); the number of nodes in the intermediate layer after the ResNet and the ViT backbone (128; 64, 128, 256, and 512 were tested).

## Results

### Automatic labels

Meaningful concepts can be extracted from pathology reports without the need for human intervention and can be adopted as weak labels, dramatically reducing the time needed to collect labels.

The performance of SKET (a tool to extract weak labels from reports) is evaluated on the training partition of the three use cases because SKET is a ruled-based algorithm that does not require any training. The extracted concepts are compared with the manual labels provided by medical experts. Table 4 summarizes the results. SKET reaches a weighted F1-score over 0.944 on every use case, considering the cumulative testing partition. On the single pathology workflows, the lowest performance is reached considering the Catania testing partition of CD data (0.860). Otherwise, the algorithm reaches high-level performance, always over 0.960 in terms of F1-score. SKET shows high performance in terms of concept extraction, limiting the errors to a specific type of reports where several details not related to a class are reported. These reports include low-level details of the tissue structures, but also annotations about family history, about past exams, about immunohistochemistry findings. These reports are usually wordy, include multiple sections, but represent a small minority of the whole dataset. Therefore, SKET shows high performance when tested on more concise reports, including only information about the histopathological findings.

**Table 4:** Overview of SKET's performance on extracting meaningful concepts from pathology reports, evaluated in terms of F1-score. The performance is evaluated comparing the concepts extracted by SKET as labels and the ground-truth labels. The algorithm is evaluated considering the training partitions of three use cases (celiac disease, lung cancer, and colon cancer), because SKET requires no training. The results are assessed based on data from Catania and RUMC and their combination for every tissue use case.

Use case	Catania	RUMC	Cumulative
Celiac disease	0.860	0.964	0.944
Lung cancer	0.969	0.975	0.976
Colon cancer	0.976	0.961	0.971

SKET can be adopted to extract labels from unlabeled datasets and annotate large amounts of data that can be used to train DL models. Table 5 summarizes the results. When tested on a Tesla V100 GPU, SKET requires between 0.006 (ceiling annotation time) and 0.03 (floor annotation time) seconds to extract concepts from a report, depending on its length. A human expert needs between 10 s (ceiling annotation time) and 30 s (floor annotation time) to extract concepts from a report, depending on its length and content. Considering the worst-case scenario for SKET and the best-case scenario for a human expert (0.03 s vs. 10 s), the algorithm is still around 333 times (0.03/10) faster than a human. For instance, in the best-case scenario, the weak labeling of 10,000 WSIs would require 300,000 s (around 83 h, without breaks) for human experts; in the worst-case scenario, it would require 300 s (5 min) to SKET. Therefore, the application of SKET leads to save 99.7% of time required in comparison with human experts. Even considering an unreasonable effectiveness of a human experts, such as 1 s per iteration, would lead to save 97% of the time. A detail relevant to stress is that the comparison considers the best possible condition for a human expert (no breaks, no wasted time, and ceiling performance) and the worst condition for SKET (floor performance).

**Table 5:** Overview of the time needed by SKET and a human expert to annotate reports. The comparison involves three possible durations for a human expert and two for SKET. The values chosen for a human expert are 1 s, 10 s, and 30 s, respectively, extremely (but unfeasible) fast annotators, ceiling of the annotation range, and floor of the annotation range. The values chosen for SKET are 0.006 s and 0.03 s, respectively, with the ceiling of the annotation range and the floor of the annotation range. The comparison is made considering 10,000 annotated reports and includes the percentage of time saved.

Time per iteration	Automatic (min)	Manual (min)	Percentage saved
A: 0.006 s/M: 1 s	1	166.6666667	99.40%
A: 0.03/M: 1 s	5	166.6666667	97.00%
A: 0.006 s/M: 10s	1	1666.666667	99.94%
A: 0.03/M: 10s	5	1666.666667	99.70%
A: 0.006 s/M: 30s	1	5000	99.98%
A: 0.03/M: 30s	5	5000	99.90%

### Celiac disease

The classification performance of multiple computer vision architectures trained with binary automatically annotated data to classify CD WSIs is as effective as the performance reached by models using manually annotated data.

Table 6, Table 7 summarize the results. The highest performance using manual labels is reached using a ViT architecture (F1-score = 0.914 *±* 0.014 on the test partition), even if on the Catania partition transMIL shows the highest performance. The results are relatively similar for the three architectures. Table 6 shows the classification performance obtained using binary manual labels and noisy labels. This experiment aims to investigate general rules for the adoption of automatic labels on the binary classification of WSIs. Considering all the architectures, the performance is similar to the one obtained using manual labels, particularly when fewer than 10% of the training samples are wrongly annotated. The difference in terms of performance is not statistically significant. When the percentage of wrongly annotated training is 20% (or more) the performance is reduced and the difference, compared with manual labels, is statistically significant, suggesting that this percentage of wrongly annotated labels can be considered as a threshold for the adopting of automatic weak labels in a binary classification scenario. Table 7 compares automatic labels generated with SKET and manual labels. The comparison among automatic and manual labels shows an F1-score equal to 0.944, suggesting that the algorithm should lead to performance similar to the one obtained with noisy labels when the percentage of mislabeled data is between 2% and 5%. The results confirm the hypothesis because the performance is slightly worse than the one obtained using manual labels, but the gap is not statistically significant (according to the Wilcoxon Rank-Sum test, comparing every setup to the one where manual labels are used), showing the effectiveness of automatic labels in a binary classification scenario.

**Table 6:** Results on the classification of celiac disease, in terms of F1-score. The performance is evaluated considering three computer vision architectures: CLAM, transMIL, and ViT. The architectures are trained with manual weak binary labels and with noisy, weak labels, randomly perturbed with different percentages of noise. The percentage of noisy labels is reported in the ‘noisy labels' column, whereas the quality of the labels is reported in terms of F1-score, ‘F1 labels' column. The goal is to evaluate the effect that noisy weak labels have on the binary classification of WSIs. For every setup, the F1-score average and standard deviation of the classification performance are reported, considering the models trained with the 10-fold cross-validation. The setups where a difference that is statistically significant in terms of performance (compared with the models trained with manual labels) are marked with an asterisk (*).

Noisy labels	F1 labels	Model	Catania	RUMC	Cumulative
Manual	–	CLAM SB	0.958 *±* 0.009	0.846 *±* 0.023	0.900 *±* 0.012
		transMIL	0.968 *±* 0.009	0.850 *±* 0.019	0.906 *±* 0.010
		ViT	0.953 *±* 0.011	0.877 *±* 0.021	0.914 *±* 0.014
1%	0.977	CLAM SB	0.954 *±* 0.016	0.849 *±* 0.024	0.900 *±* 0.018
		transMIL	0.968 *±* 0.009	0.864 *±* 0.010	0.914 *±* 0.007
		ViT	0.954 *±* 0.014	0.896 *±* 0.019	0.925 *±* 0.010
2%	0.968	CLAM SB	0.951 *±* 0.012	0.873 *±* 0.021	0.911 *±* 0.014
		transMIL	0.965 *±* 0.011	0.853 *±* 0.021	0.907 *±* 0.010
		ViT	0.944 *±* 0.017	0.877 *±* 0.021	0.910 *±* 0.013
5%	0.933	CLAM SB	0.951 *±* 0.019	0.862 *±* 0.019	0.905 *±* 0.017
		transMIL	0.958 *±* 0.012*	0.857 *±* 0.018	0.905 *±* 0.011
		ViT	0.938 *±* 0.026	0.880 *±* 0.026	0.910 *±* 0.020
10%	0.909	CLAM SB	0.952 *±* 0.013	0.862 *±* 0.023	0.905 *±* 0.017
		transMIL	0.953 *±* 0.026*	0.838 *±* 0.033	0.893 *±* 0.027
		ViT	0.957 *±* 0.014	0.860 *±* 0.023	0.906 *±* 0.014
20%	0.804	CLAM SB	0.922 *±* 0.026*	0.819 *±* 0.029	0.869 *±* 0.023*
		transMIL	0.933 *±* 0.024*	0.822 *±* 0.013*	0.875 *±* 0.016*
		ViT	0.925 *±* 0.017*	0.834 *±* 0.025*	0.879 *±* 0.017*
50%	0.566	CLAM SB	0.537 *±* 0.228*	0.450 *±* 0.081*	0.490 *±* 0.145*
		transMIL	0.765* *±* 0.097*	0.502* *±* 0.02*	0.633 *±* 0.041*
		ViT	0.440 *±* 0.302*	0.459 *±* 0.029*	0.480 *±* 0.141*

**Table 7:** Results on the classification of celiac disease, in terms of F1-score. The performance is evaluated considering three computer vision architectures: CLAM, transMIL, and ViT. The architectures are trained with automatic and manual weak binary labels generated by extracting meaningful concepts from the corresponding pathology report using the SKET algorithm. The performance of SKET is reported in the ‘noisy labels' column. The goal is to evaluate the effectiveness of automatic labels on the binary classification of WSIs. For every setup, the F1-score average and standard deviation of the classification performance are reported, considering the models trained with the 10-fold cross-validation. The setups where the difference is statistically significant in terms of performance (compared with the models trained with manual labels) are marked with an asterisk (*).

Noisy labels	F1 labels	Model	Catania	RUMC	Cumulative
Automatic	0.944	CLAM SB	0.948 *±* 0.015	0.857 *±* 0.017	0.901 *±* 0.013
		transMIL	0.960 *±* 0.012	0.845 *±* 0.017	0.900 *±* 0.014
		ViT	0.938 *±* 0.023	0.889 *±* 0.024	0.915 *±* 0.015
Manual	–	CLAM SB	0.958 *±* 0.009	0.846 *±* 0.023	0.900 *±* 0.012
		transMIL	0.968 *±* 0.009	0.85 *±* 0.019	0.906 *±* 0.010
		ViT	0.953 *±* 0.011	0.877 *±* 0.021	0.914 *±* 0.014

### Lung cancer

The classification performance of multiple computer vision architectures trained with automatically annotated multiclass data to classify lung cancer WSIs is as effective as the performance reached by models using manually annotated data.

Table 8, Table 9 summarize the results. The highest performance using manual labels is reached using a ViT architecture (F1-score = 0.763 *±* 0.012) on both test partitions, dramatically outperforming the other two architectures (CLAM reaches 0.674 *±* 0.016, whereas transMIL reaches 0.696 *±* 0.016). Table 8 shows the classification performance obtained using multiclass manual labels and noisy labels. This experiment aims to investigate general rules for the adoption of automatic labels on the multiclass classification of WSIs. Considering all the architectures, the performance is similar to the one obtained using manual labels, especially until 20% of training samples are wrongly-annotated, the difference in terms of performance is not statistically significant. When the percentage of wrongly annotated training is 50% the performance degrades and the difference, compared with manual labels, is statistically significant, suggesting this percentage of wrongly annotated labels can be considered as a threshold for the adoption of automatic weak labels in a multiclass classification scenario. Table 9 shows the performance of automatic labels and manual labels. This comparison represents a real scenario of automatic data labeling, where automatic labels are generated by extracting concepts from reports. The comparison among labels shows an F1-score equal to 0.976, suggesting that the algorithm should lead to a performance similar to the one obtained in the previous experiment using 2% and 5%. The results confirm the hypothesis because the performance is slightly worse than the one obtained using manual labels, but the gap is not statistically significant (according to the Wilcoxon Rank-Sum test, comparing every setup to the one where manual labels are used).

**Table 8:** Results on the classification of lung cancer, in terms of F1-score. The performance is evaluated considering three computer vision architectures: CLAM, transMIL, and ViT. The architectures are trained with manual weak multiclass labels and with noisy weak labels, randomly perturbed according to different percentages of noise. The percentage of noisy labels is reported in the ‘noisy Labels' column, whereas the accuracy of the labels is reported in terms of F1-score, ‘F1 labels' column. The goal is to evaluate the effect that noisy weak labels have on the multiclass classification of WSIs. For every setup, the F1-score average and standard deviation of the classification performance are reported, considering the models trained with the 10-fold cross-validation. The setups where the difference is statistically significant in terms of performance (compared with the models trained with manual labels) are marked with an asterisk (*).

Noisy labels	F1 labels	Model	Catania	RUMC	Cumulative
Manual	–	CLAM MB	0.617 *±* 0.027	0.717 *±* 0.023	0.674 *±* 0.016
		transMIL	0.635 *±* 0.024	0.745 *±* 0.024	0.696 *±* 0.016
		ViT	0.705 *±* 0.033	0.812 *±* 0.02	0.763 *±* 0.012
1%	0.991	CLAM MB	0.624 *±* 0.022	0.725 *±* 0.021	0.681 *±* 0.014
		transMIL	0.634 *±* 0.042	0.756 *±* 0.012	0.700 *±* 0.020
		ViT	0.697 *±* 0.035	0.817 *±* 0.018	0.762 *±* 0.021
2%	0.98	CLAM MB	0.621 *±* 0.034	0.721 *±* 0.016	0.677 *±* 0.018
		transMIL	0.642 *±* 0.033	0.739 *±* 0.011	0.695 *±* 0.017
		ViT	0.698 *±* 0.032	0.807 *±* 0.026	0.757 *±* 0.026
5%	0.957	CLAM MB	0.609 *±* 0.035	0.715 *±* 0.022	0.670 *±* 0.021
		transMIL	0.622 *±* 0.050	0.743 *±* 0.015	0.687 *±* 0.026
		ViT	0.699 *±* 0.027	0.809 *±* 0.029	0.758 *±* 0.020
10%	0.907	CLAM MB	0.601 *±* 0.037	0.690 *±* 0.034	0.653 *±* 0.027
		transMIL	0.615 *±* 0.029	0.739 *±* 0.025	0.683 *±* 0.023
		ViT	0.699 *±* 0.026	0.808 *±* 0.018	0.757 *±* 0.015
20%	0.822	CLAM MB	0.579 *±* 0.060	0.725 *±* 0.038	0.658 *±* 0.042
		transMIL	0.614 *±* 0.039	0.743 *±* 0.017	0.684 *±* 0.018
		ViT	0.702 *±* 0.018	0.808 *±* 0.015	0.759 *±* 0.012
50%	0.561	CLAM MB	0.409 *±* 0.087*	0.528 *±* 0.069*	0.477 *±* 0.065*
		transMIL	0.483 *±* 0.055*	0.566 *±* 0.027*	0.537 *±* 0.031*
		ViT	0.576 *±* 0.049*	0.701 *±* 0.040*	0.643 *±* 0.038*

**Table 9:** Results on the classification of lung cancer, in terms of F1-score. The performance is evaluated considering three computer vision architectures: CLAM, transMIL, and ViT. The architectures are trained with automatic and manual weak multiclass labels, generated by extracting meaningful concepts from the corresponding pathology report using the SKET algorithm. The performance of SKET is reported in the ‘noisy labels' column. The goal is to evaluate the effectiveness of automatic labels on the multiclass classification of WSIs. For every setup, the F1-score average and standard deviation of the classification performance are reported, considering the models trained with the 10-fold cross-validation. The setups where the difference is statistically significant in terms of performance (compared with the models trained with manual labels) are marked with an asterisk (*).

Noisy labels	F1 labels	Model	Catania	RUMC	Cumulative
Automatic	0.976	CLAM MB	0.623 *±* 0.031	0.705 *±* 0.028	0.67 *±* 0.020
		transMIL	0.620 *±* 0.027	0.740 *±* 0.027	0.686 *±* 0.018
		ViT	0.682 *±* 0.041	0.820 *±* 0.014	0.756 *±* 0.022
Manual	–	CLAM SB	0.617 *±* 0.027	0.717 *±* 0.023	0.674 *±* 0.016
		transMIL	0.635 *±* 0.024	0.745 *±* 0.024	0.696 *±* 0.016
		ViT	0.705 *±* 0.033	0.812 *±* 0.020	0.763 *±* 0.012

### Colon cancer

The classification performance of multiple computer vision architectures trained with multilabel automatically annotated data to classify colon cancer WSIs is as effective as the performance reached by models using manually annotated data.

Table 10, Table 11 summarize the results. The highest performance using manual labels is reached using a ViT architecture (F1-score = 0.831 *±* 0.009) on both test partitions, outperforming the other two architectures (CLAM reaches 0.773 *±* 0.015, whereas transMIL reaches 0.791 *±* 0.008). Table 10 shows the classification performance obtained using multilabel manual labels and noisy labels. This experiment aims to investigate general rules for the adoption of automatic labels on the multilabel classification of WSIs. Considering all the architectures, the performance is similar to the one obtained using manual labels, especially until 20% of training samples are wrongly annotated, the difference in terms of performance is not statistically significant. When the percentage of wrongly annotated training is 50% the performance degrades and the difference, compared with manual labels, is statistically significant, suggesting this percentage of wrongly annotated labels can be considered as a threshold for the adoption of automatic weak labels in a multilabel classification scenario. Table 11 includes the comparison of automatic labels and manual labels. This comparison represents a real scenario of automatic data labeling, where automatic labels are generated by extracting concepts from reports. The comparison among labels shows an F1-score equal to 0.971, suggesting that the algorithm should lead to a performance similar to the one obtained in the previous experiment using 2% and 5%. The results confirm the hypothesis, because the performance is slightly worse than the one obtained using manual labels, but the gap is not statistically significant (according to the Wilcoxon Rank-Sum test, comparing every setup to the one where manual labels are used).

**Table 10:** Results on the classification of colon cancer, in terms of F1-score. The performance is evaluated considering three computer vision architectures: CLAM, transMIL, and ViT. The architectures are trained with manual weak multilabel labels and with noisy weak labels, randomly perturbed according to different percentages of noise. The percentage of noisy labels is reported in the ‘noisy labels' column, whereas the accuracy of the labels is reported in terms of F1-score, ‘F1 labels' column. The goal is to evaluate the effect that noisy weak labels have on the multilabel classification of WSIs. For every setup, the F1-score average and standard deviation of the classification performance are reported, considering the models trained with the 10-fold cross-validation. The setups where the difference is statistically significant in terms of performance (compared with the models trained with manual labels) are marked with an asterisk (*).

Noisy labels	F1 labels	Model	Catania	RUMC	Cumulative
Manual	–	CLAM MB	0.761 *±* 0.015	0.780 *±* 0.017	0.773 *±* 0.015
		transMIL	0.771 *±* 0.015	0.807 *±* 0.007	0.791 *±* 0.008
		ViT	0.824 *±* 0.016	0.837 *±* 0.007	0.831 *±* 0.009
1%	0.988	CLAM MB	0.761 *±* 0.018	0.776 *±* 0.016	0.771 *±* 0.015
		transMIL	0.772 *±* 0.014	0.810 *±* 0.009	0.793 *±* 0.010
		ViT	0.827 *±* 0.018	0.835 *±* 0.005	0.831 *±* 0.009
2%	0.978	CLAM MB	0.745 *±* 0.018	0.764 *±* 0.019	0.757 *±* 0.017
		transMIL	0.777 *±* 0.019	0.807 *±* 0.010	0.793 *±* 0.012
		ViT	0.821 *±* 0.019	0.837 *±* 0.005	0.831 *±* 0.009
5%	0.943	CLAM MB	0.765 *±* 0.018	0.771 *±* 0.021	0.769 *±* 0.018
		transMIL	0.766 *±* 0.013	0.808 *±* 0.009	0.790 *±* 0.008
		ViT	0.819 *±* 0.015	0.835 *±* 0.008	0.828 *±* 0.009
10%	0.898	CLAM MB	0.767 *±* 0.023	0.777 *±* 0.019	0.774 *±* 0.018
		transMIL	0.768 *±* 0.017	0.805 *±* 0.009	0.789 *±* 0.010
		ViT	0.827 *±* 0.015	0.836 *±* 0.005	0.833 *±* 0.008
20%	0.814	CLAM MB	0.748 *±* 0.026	0.757 *±* 0.020	0.754 *±* 0.019
		transMIL	0.772 *±* 0.012	0.809 *±* 0.010	0.793 *±* 0.008
		ViT	0.822 *±* 0.020	0.833 *±* 0.003	0.829 *±* 0.009
50%	0.587	CLAM MB	0.697 *±* 0.042*	0.646 *±* 0.086*	0.670 *±* 0.056*
		transMIL	0.723 *±* 0.027*	0.720 *±* 0.024*	0.721 *±* 0.015*
		ViT	0.811 *±* 0.016*	0.804 *±* 0.021*	0.807 *±* 0.016*

**Table 11:** Results on the classification of colon cancer, in terms of F1-score. The performance is evaluated considering three computer vision architectures: CLAM, transMIL, and ViT. The architectures are trained with automatic and manual weak multilabel labels, generated by extracting meaningful concepts from the corresponding pathology report using the SKET tool. The performance of SKET is reported in the ‘noisy labels' column. The goal is to evaluate the effectiveness of automatic labels on the multilabel classification of WSIs. For every setup, the classification performance in terms of F1-score average and the standard deviation are reported, considering the models trained with the 10-fold cross-validation. The setups where the difference is statistically significant in terms of performance (compared with the models trained with manual labels) are marked with an asterisk (*).

Noisy labels	F1 labels	Model	Catania	RUMC	Cumulative
Automatic	0.971	CLAM MB	0.761 *±* 0.014	0.771 *±* 0.019	0.767 *±* 0.016
		transMIL	0.759 *±* 0.013	0.801 *±* 0.004	0.783 *±* 0.005
		ViT	0.813 *±* 0.014	0.836 *±* 0.008	0.826 *±* 0.008
Manual	–	CLAM MB	0.761 *±* 0.015	0.780 *±* 0.017	0.773 *±* 0.015
		transMIL	0.771 *±* 0.015	0.807 *±* 0.007	0.791 *±* 0.008
		ViT	0.824 *±* 0.016	0.837 *±* 0.007	0.831 *±* 0.009

## Discussion

This paper evaluates the application of weak automatic labels to train computer algorithms on classification, adding noise to see how tolerant these models are to errors that can happen with a fully automatic label extraction. The application of automatic weak labels can dramatically reduce the time needed to collect samples to train algorithms for the analysis of biomedical data. However, it is not clear under which conditions automatic labels can be adopted to train algorithms and what the minimum quality of these labels needs to be.

The results achieved in the paper show that automatic labels are as effective as manual labels for the classification of WSIs. The first experiments (where manual labels are compared to different percentages of noisy labels) allow to identify patterns in the algorithm performance. The noise introduced by mislabeled samples (inherently present within automatically extracted labels) impacts the classification accuracy and robustness. The model can compensate for the effect of mislabeled samples on the training using the manual labels until a fixed percentage of mislabeled data: 10% regarding CD (binary labels) and 20% regarding lung and colon cancer (respectively, multiclass and multilabel labels). This performance decrease can be explained by considering the different natures of labels. Mislabeled samples have a high impact on binary classification because the label flipping leads to opposite results. Annotation errors are also disruptive in multiclass labels, even if, in this case, the effect can be smoothed if the errors involve similar classes (already prone to uncertainty). Another explanation for this gap can be identified in the training dataset size. A relevant parameter to consider when automatic labels are applied is the size of the training dataset because the effect of mislabeled samples on the training may be compensated by the other samples. In this paper, the CD training dataset includes around 1000 samples, whereas the lung cancer dataset includes around 2500 samples, and the colon cancer one includes around 6500. In the CD use case, when the percentage of mislabeled samples is 20% or more, the performance of the architectures is no longer comparable with the one reached using manual labels when the percentage of mislabeled samples is 20%. This result suggests that automatic labels can be adopted when the algorithm used to generate them is reasonably accurate. The effect of noisy labels can also be identified in the performance standard deviation: the higher the percentage of noisy labels, the less robust the three architectures are.

The architectures trained using automatic labels reach a performance comparable (i.e., the performance difference is not statistically significant) with the one reached using manual labels. The results are consistent among different tissue types (i.e., celiac, lung, and colon images), architectures and type of problem to solve (i.e., binary, multiclass, and multilabel classification). The conditions identified using randomly perturbed noisy data are also tested on a real case scenario, where the automatic labels are generated using SKET, an NLP algorithm to extract meaningful concepts from pathology reports. The results obtained using SKET to generate automatic weak labels are consistent with what obtained with randomly perturbed labels. SKET leads to a percentage of mislabeling between 2% and 5%. The models trained to analyze WSIs using labels provided by SKET reached similar performance with the ones trained using both 2% and 5% of mislabeled samples. The application of SKET has been designed to offer a real-case scenario for the application of automatic labels for the training of DL algorithms. However, it helps also to assess that the percentage of noise is the main factor to consider when considering automatic labeled data. Both SKET and the random perturbation of ground-truth labels provide mislabeled samples, but the distribution of errors that SKET can make is concentrated on a specific type of report with certain characteristics, whereas random perturbation of labels usually leads to a more uniform possibility of errors. However, the performance on the image classification is comparable (the difference is not statistically significant), implying that the nature of possible annotation errors is only a factor to consider according to the percentage of errors that can be tolerated by DL algorithms trained to analyze images.

The fact that automatic labels are as effective as manual labels opens many perspectives for the computational pathology domain and for the biomedical domain in general. Automatic labels limit the need for medical experts to annotate data, which can save up to 99.7% of time otherwise needed to annotate reports in order to infer labels. Therefore, a dataset that includes around 10,000 documents can be weakly annotated in around 5 min and as the results show the quality of automatic annotation is high enough for training the models. Considering that a large amount of biomedical data is produced every year and only a small percentage is annotated, this allows the exploitation of a vast amount of data that can be used to build more accurate and robust models while still guaranteeing robust performance, helping medical experts with tools to diagnose diseases more effectively. The implementation details, such as architecture, task, and pre-processing techniques adopted in this research, can be tailored to fit the specific characteristics of another problem.

## Conclusions

The application of automatic labels may help to exploit the vast amounts of unlabeled biomedical samples to train more robust models, reducing by 99.7% the time needed to collect weakly annotated samples. However, it is still unclear when this label is effective. This paper evaluates the performance of different percentages of noisy labels (1%, 2%, 5%, 10%, 20%, and 50%) and compares the results with the performance obtained by the same architectures but using manual weak labels provided by medical experts. After some rules are identified (e.g., training datasets with 10% of mislabeled samples lead to performance comparable to that obtained using manual labels), SKET, an algorithm for extracting meaningful concepts from reports, is used to generate automatic weak labels. The performance reached by the models trained with SKET labels is comparable (no statistically significant difference) to the one obtained with manual labels, showing the effectiveness of automatic labels. The result can allow for the annotation of samples contained in hospitals without the need for human effort, paving the way for increasingly accurate algorithms. The code, including implementing the computer vision algorithms to classify WSIs, is publicly available on GitHub (https://github.com/ilmaro8/wsi analysis).

## Declaration of competing interest

The authors declare that they have no known competing financial interests or personal relationships that could have appeared to influence the work reported in this paper.

## References

[1] Abels E., Pantanowitz L., Aeffner F. (2019). Computational pathology definitions, best practices, and recommendations for regulatory guidance: a white paper from the digital pathology association. J Pathol..

[2] Benson A.B., Venook A.P., Al-Hawary M.M. (2018). Nccn guidelines insights: colon cancer, version 2.2018. J Natl Compr Cancer Netw..

[3] Buslaev A., Iglovikov V.I., Khvedchenya E., Parinov A., Druzhinin M., Kalinin A.A. (2020). Albumentations: fast and flexible image augmentations. Information.

[4] Caio G., Volta U., Sapone A. (2019). Celiac disease: a comprehensive current review. BMC Med..

[5] Campanella G., Hanna M.G., Geneslaw L. (2019). Clinical-grade computational pathology using weakly supervised deep learning on whole slide images. Nat Med..

[6] Carbonneau M.A., Cheplygina V., Granger E., Gagnon G. (2018). Multiple instance learning: a survey of problem characteristics and applications. Pattern Recogn..

[7] Chen R.J., Chen C., Li Y. (2022). Proceedings of the IEEE/CVF Conference on Computer Vision and Pattern Recognition.

[8] Chen T., Kornblith S., Norouzi M., Hinton G. (2020). International Conference on Machine Learning.

[9] Cifci D., Veldhuizen G.P., Foersch S., Kather J.N. (2023). AI in computational pathology of cancer: improving diagnostic workflows and clinical outcomes?. Annu Rev Cancer Biol..

[10] De Matos J., Ataky S.T.M., de Souza Britto A., Soares de Oliveira L.E., Lameiras Koerich A. (2021). Machine learning methods for histopathological image analysis: a review. Electronics.

[11] Deng S., Zhang X., Yan W. (2020). Deep learning in digital pathology image analysis: a survey. Front Med..

[12] Ferrıs L.B., Püttmann S., Marini N. (2024). Medical Imaging 2024: Digital and Computational Pathology.

[13] Fraggetta F., Garozzo S., Zannoni G.F., Pantanowitz L., Rossi E.D. (2017). Routine digital pathology workflow: the Catania experience. J Pathol Inform..

[14] Fraggetta F., Limperio V., Ameisen D. (2021). Best practice recommendations for the implementation of a digital pathology workflow in the anatomic pathology laboratory by the European Society of Digital and Integrative Pathology (ESDIP). Diagnostics.

[15] Goode A., Gilbert B., Harkes J., Jukic D., Satyanarayanan M. (2013). Openslide: a vendor-neutral software foundation for digital pathology. J Pathol Inform..

[16] Gurcan M.N., Boucheron L.E., Can A., Madabhushi A., Rajpoot N.M., Yener B. (2009). Histopathological image analysis: a review. IEEE Rev Biomed Eng..

[17] Han K., Wang Y., Chen H. (2020). A survey on visual transformer. arXiv preprint.

[18] Hanna M.G., Reuter V.E., Ardon O. (2020). Validation of a digital pathology system including remote review during the COVID-19 pandemic. Mod Pathol..

[19] Hashimoto N., Fukushima D., Koga R. (2020). Proceedings of the IEEE/CVF Conference on Computer Vision and Pattern Recognition.

[20] Hewer E. (2020). The oncologist’s guide to synoptic reporting: a primer. Oncology.

[21] Ilse M., Tomczak J., Welling M. (2018). International Conference on Machine Learning.

[22] Janowczyk A., Zuo R., Gilmore H., Feldman M., Madabhushi A. (2019). Histoqc: an open-source quality control tool for digital pathology slides. JCO Clin Cancer Inform..

[23] Junczys-Dowmunt M., Grundkiewicz R., Dwojak T. (2018). Marian: fast neural machine translation in c++. arXiv preprint.

[24] Karimi D., Dou H., Warfield S.K., Gholipour A. (2020). Deep learning with noisy labels: exploring techniques and remedies in medical image analysis. Med Image Anal..

[25] Krupinski E.A., Graham A.R., Weinstein R.S. (2013). Characterizing the development of visual search expertise in pathology residents viewing whole slide images. Hum Pathol..

[26] Van der Laak J., Litjens G., Ciompi F. (2021). Deep learning in histopathology: the path to the clinic. Nat Med..

[27] Lebwohl B., Rubio-Tapia A. (2021). Epidemiology, presentation, and diagnosis of celiac disease. Gastroenterology.

[28] Litjens G., Ciompi F., van der Laak J. (2022). A decade of gigascience: the challenges of gigapixel pathology images. GigaScience.

[29] Lu M.Y., Williamson D.F., Chen T.Y., Chen R.J., Barbieri M., Mahmood F. (2021). Data-efficient and weakly supervised computational pathology on whole-slide images. Nat Biomed Eng..

[30] Madabhushi A., Lee G. (2016). Image analysis and machine learning in digital pathology: challenges and opportunities. Med Image Anal..

[31] Marchesin S., Giachelle F., Marini N. (2022). Empowering digital pathology applications through explainable knowledge extraction tools. J Pathol Inform..

[32] Marini N., Atzori M., Ot’alora S., Marchand-Maillet S., Müller H. (2021). Proceedings of the IEEE/CVF International Conference on Computer Vision.

[33] Marini N., Marchesin S., Ot’alora S. (2022). Unleashing the potential of digital pathology data by training computer-aided diagnosis models without human annotations. NPJ Digi Med..

[34] Marini N., Marchesin S., Wodzinski M. (2024). Multimodal representations of biomedical knowledge from limited training whole slide images and reports using deep learning. Med Image Anal..

[35] Marini N., Ot’alora S., Podareanu D. (2021). Multi scale tools: a python library to exploit multi-scale whole slide images. Front Comput Sci..

[36] Marini N., Otalora S., Wodzinski M. (2023). Data-driven color augmentation for h&e stained images in computational pathology. J Pathol Inform..

[37] Menotti L., Silvello G., Atzori M. (2023). Modelling digital health data: the examode ontology for computational pathology. J Pathol Inform..

[38] Merchant F., Castleman K. (2022).

[39] Mikolov T., Sutskever I., Chen K., Corrado G.S., Dean J. (2013). Distributed representations of words and phrases and their compositionality. Adv Neural Inform Process Syst..

[40] Neumann M., King D., Beltagy I., Ammar W. (2019). Scispacy: fast and robust models for biomedical natural language processing. arXiv preprint.

[41] Oquab M., Darcet T., Moutakanni T. (2023). Dinov2: learning robust visual features without supervision. arXiv preprint.

[42] Organization, W.H (2023). Lung Cancer. https://www.who.int/news-room/fact-sheets/detail/lung-cancer.

[43] Püttmann S., Ferris L.B., Marini N. (2024). Medical Imaging 2024: Computer-Aided Diagnosis.

[44] Schabath M.B., Cote M.L. (2019). Cancer progress and priorities: lung cancer. Cancer Epidemiol Biomarkers Prev..

[45] Shao Z., Bian H., Chen Y. (2021). Transmil: transformer based correlated multiple instance learning for whole slide image classification. Adv Neural Inf Proces Syst..

[46] Sharir G., Noy A., Zelnik-Manor L. (2021). An image is worth 16x16 words, what is a video worth?. arXiv preprint.

[47] Travis W.D. (2011). Pathology of lung cancer. Clin Chest Med..

[48] Vaswani A., Shazeer N., Parmar N. (2017). Attention is all you need. Adv Neural Inf Proces Syst..

[49] Wang Y., Li J., Metze F. (2019). ICASSP 2019–2019 IEEE International Conference on Acoustics, Speech and Signal Processing (ICASSP).

[50] Woolson R.F. (2007). Wiley Encyclopedia of Clinical Trials.

[51] Xu H., Xu Q., Cong F. (2023). Vision transformers for computational histopathology. IEEE Rev Biomed Eng.

